# Mr. Cole's Case of Tetanus

**Published:** 1802-01

**Authors:** T. Comyns Cole

**Affiliations:** Woodstock Street, New Bond Street


GENTLEMEN,

THE following Caſe of Tetanus ſuccesſfully treated by the exhibition of Opium, if worthy a place in your uſeful Miſcellany, will not I hope altogether prove uſeleſs to the generality of your readers, independently of its being attended with ſome adventitious circumſtances.

*Woodstock Street, New Bond Street,* I am, &c.

*December* 10, 1801. T. COMYNS COLE.

---

I was deſired by Sir John Lade, to ſee his coachman's wife, Mary Carter, ætat. twenty-two, of a ſpare thin habit, rather of a delicate conſtitution, and frequently ſubject to rheumatiſm. I ſaw her Oct. 2, 1801, in the morning; the ſtate I found her in was as follows. Her countenance pale and contracted, a rigidity of the neck, with the head ſtrongly pulled backwards, her jaws completely locked, cold damp ſweats, particularly on the face and hands; her pulſe 130, irregular and ſmall, with great proſtration of ſtrength; ſhe could not bear to be moved in her bed, in which ſhe had been confined for ſix days, without taking the leaſt nouriſhment, and a child of eight months old at her breaſt, within two days of the time I ſaw her. From theſe circumſtances, and the great exhauſtion that had taken place, joined with the diſeaſe, I conſidered it a hopeleſs caſe. The tetanic affection had been coming on gradually for a fortnight, proceeding from a cold, caught by ſleeping in a damp room; ſhe frequently felt in the beginning of the attack, a violent pain at the lower end of the *sternum*, *from thence darting into the back*; a ſtiffneſs and uneaſineſs about the neck, which ſhe ſuppoſed to be what is vulgarly called, a *crick in the neck*; the muſcles of the jaw were affected with a ſpaſtic rigidity, which ſhe firſt diſcovered by eating an apple; but there was no pain in the root of the tongue, or any difficulty in ſwallowing, ſhe ſpoke without uneaſineſs, and during the whole of the diſeaſe the external muſcles of the abdomen were not affected with ſpaſm, though ſhe had not a ſtool for four days.

The firſt thing I ordered was a laxative clyſter with ſap. mol. ℥iij. which brought away ſome hard fæces; after that ſhe had another clyſter with tinct. opii ʒiij. mixed with eight ounces of mutton broth; it remained four hours, and at the expiration of that time, her mouth opened ſufficiently to admit a tea ſpoon in it, and to take nouriſhment in ſmall quantities often often repeated; I ordered two grains of *opium purif.* to be given her every two hours, *durante nocte*, with nourifhment and wine at proper intervals; fhe paft a quiet night, *but did not sleep*; the next morning her mouth was nearly half open, her countenance looked better, her pulfe was lefs frequent, more regular, and of a greater degree of ftrength; I continued the two grains of *opium purif.* every two hours, which *she swallowed very well*; in the evening fhe feemed uneafy, fweated profufely, and complained of a pain in the lower end of the fternum, darting into the back, attended with fhortnefs of breath, and a pulfe irregular and hurried. As I was afraid the *tetanic spasms* might come on in the night, attended with the fpaftic rigidity of the jaw, I ordered her a pill of *six grains of opium purif.* to be given at bed time; and to keep up the *influence* of it, I repeated *two grains* every two hours with a cordial draught. The next morning I found her better, though fhe had little fleep during the night, but felt as fhe expreffed herfelf *happy and comfortable*, her head quite clear and free from pain, notwithftanding fhe took in the laft twenty-four hours *thirty grains of opium purif.* which may be confidered equal to *six hundred drops* of the *tincture.*

As a week had now elapfed without her having a ftool, (excepting by clyfters,) I thought it expedient to procure one by giving her a mixture of *ol. ricini* (omitting the opium,) a fourth part to be taken every two hours with a pill of *ext. colocy. comp. et calomel.* Eight hours elapfed before fhe had a motion, which was very coftive, and fhe had three afterwards more lax; during that night *the pain at the lower end of the sternum* came on with violent fpafms in the mufcles of the tongue, which was forced out between the teeth, and much injured by being frequently bitten; and in the morning when I faw her, the jaw was again *nearly locked.* I attributed the return of the fpafms to the omiffion of the opium for *ten hours*; I therefore ordered again a clyfter with tinct. opii ʒiij. and continued the two grains of opium purif. every two hours with the cordial draught, and frequent ufe of nourifhment and wine; in the evening fhe was better, free from fpafms and pain; the jaw more open. She continued the medicines *durante nocte*; the next morning her mouth was half open; fhe paffed a good night, but had fweated *profusely.* I continued the opium in the fame quantity every *three* hours for four days, and at the expiration of that time, I perceived a thick miliary eruption upon the arms and neck, and a few on the face, with fmall white veficles; her breafts were now become painful and diftended, the child not having fucked her for *thirteen days*; I therefore defired her breafts to be flightly drawn, and rubbed with the linim linim. ammon. and from that time her milk gradually left her; her pulfe was fmall and quick, but regular; I thought it neceffary, after giving her fome opening medicine, (which procured a ftool without a return of the fpafms) to give her the ext. cinch. joined with two grains of opium every three hours, leffening *the latter by degrees*, till fhe wholly difcontinued it; but the *former* fhe took regularly, until her ftrength was completely reftored, and every fymptom of tetanic affection entirely overcome; fhe is now as well as fhe ever was in her life.

The above Cafe, in my opinion, fully evinces the efficacy of opium *in large and repeated doses*, fo as to keep the patient, in this difeafe, under the continual influence of it, while any difpofition remains to tetanic rigidity of the jaw. I fhall take another opportunity of making further Obfervations on this Cafe, with fome general remarks on the effects of opium in this and other difeafes.

---

